# Google Trends Analysis of Otologic Symptom Searches Following COVID-19

**DOI:** 10.22038/IJORL.2024.75617.3532

**Published:** 2024-05

**Authors:** Joshua K. Kim, Karen Tawk, Jonathan M. Kim, Hamid R. Djalilian, Mehdi Abouzari

**Affiliations:** 1 *School of Medicine, Duke University, Durham, NC, United States.*; 2 *Department of Otolaryngology-Head and Neck Surgery, University of California, Irvine, CA, United States.*; 3 *Department of Biomedical Engineering, University of California, Irvine, CA, United States.*

**Keywords:** Google Trends, Pandemic, Search Interest, Otologic Symptoms, Search Trends

## Abstract

**Introduction:**

COVID-19 infection was accompanied by otologic symptoms, a pattern that was captured early by Google Trends. The objective of this study is to investigate searches for otologic symptoms and identify correlations with the pandemic onset.

**Materials and Methods:**

Search interest for otologic symptoms was gathered using Google Trends from two years before and two years following the pandemic start date. A two-tailed Mann-Whitney U test was used to identify significant changes and effect size.

**Results:**

In total, search interest for 14 terms was collected, with significant changes identified in 11. Six terms showed increased search interest, with the most significant rises observed for headache (r=0.589, *p<*0.001), dizziness (r=0.554, *p<*0.001), and tinnitus (r=0.410, *p<*0.001). Search interest decreased for five terms, with the most notable declines found in searches for migraine headache (r=0.35, *p<*0.001) and phonophobia (r=0.22, *p=*0.002). No significant changes were seen in ear pressure (*p=*0.142), neck pain (*p=*0.935), and sudden hearing loss (*p=*0.863) searches.

**Conclusion:**

COVID-19 infection is often accompanied otologic symptoms and holds a diagnostic role. Fluctuating search interest may be attributed to a true increase in cases, media trends, or people’s desires to stay informed. Google Trends robustly captured trends in search interest and presented itself as a valuable epidemiological tool.

## Introduction

The World Health Organization declared COVID-19 a pandemic in March 2020 (1). Affected patients presented with various symptoms, including shortness of breath, muscle pain, and headaches (2). Occasionally, otolaryngological symptoms were also observed (3,4). Official guidelines soon included these symptoms, such as otalgia, tinnitus, dizziness, and aural fullness (5,6). A handful of studies have noted the association of COVID-19 with changes in hearing status and proposed various mechanisms. For example, low-frequency hearing loss was speculated to occur when the outer hair cells in the cochlea were damaged (7). Infected patients sometimes presented with sudden sensorineural hearing loss (8,9), attributed to endothelial cell dysfunction or micro-thrombosis of auditory centers due to viral infection (10,11). In some cases, vaccination was linked to auditory impairment, such as tinnitus (12). Headache symptoms were regularly observed in patients who received the vaccine, and could be linked to hearing loss (13-15). Despite these reports, establishing a definitive correlation between COVID-19 and otologic symptoms was difficult (16). External factors like feelings of loneliness, financial worries, or sadness caused by quarantining exacerbated auditory complications in patients (17). It was unclear if a causal relationship existed between COVID-19 and any auditory-related complications due to the limited scope of reports and confounding factors (5,18).

Google Trends is a public database that reports the relative interest in a search term. It has been gaining popularity in various sectors, including economics, epidemiology, and medicine (19-21). If properly used, Google Trends data provides unique insights into regional trends or events. In the United States, traffic for Lyme disease-related searches was reportedly heightened in areas where Lyme disease was endemic (20). Google Trends was also illuminating in regional cases of influenza, both in China and the United States (22,23). Google Trends is a powerful tool despite its limited documentation and its susceptibility to media trends (24,25). Google Trends showed immense potential during the pandemic. Initially, hyposmia or anosmia was infrequently charted during standard medical history taking and not identified as a symptom early in the pandemic. As COVID-19 cases rose, loss of smell was recognized as an occasional symptom. Google Trends showed a correlation between COVID-19 cases and search interest in keywords relating to loss of smell (26). Thus, trends may have been helpful in identifying loss of smell as a manifestation of COVID-19 infection well before any association was made. Tangentially related analyses in the field of otolaryngology have used Google Trends to infer how COVID-19 affected interest in otolaryngologic terms or procedures (27,28). Google Trends has shown correlations to procedural interest (29,30), with searches showing direct correlations to the timing and location of viral cases (31). Beyond correlation, search interest has proven instrumental in surveilling symptoms, serving as a barometer for public health (32). Discrete and complex patterns of COVID-19 spread and mortality were also reflected in search interest (33).

In this study, we specifically investigated reported auditory-related symptoms of COVID-19 to gain insight into whether the public experienced these symptoms or reactively sought information during the pandemic. We also wanted to understand whether any correlation existed between symptoms. No current studies have focused on the search patterns of otologic symptoms around the pandemic. Understanding which symptoms most frequently coincide with COVID-19 infection may have clinical implications, such as in detection, contact tracing, and management of COVID-19 infection.

## Materials and Methods

Google Trends was utilized to gather search data related to otologic symptoms. All “Web search” results from the United States were collected without any category restrictions, and searches with low-volume regions were excluded. This is done to minimize noise and ensure that search trends are accurate. Google Trends search interest is normalized within each region, and in low search volume regions, this process may generate noise or inaccuracies in trends, interfering with data analysis. The relative interest for searches was gathered weekly from March 11, 2018, to March 11, 2022, covering two years before and two years after the pandemic declaration by the World Health Organization (1). To construct a comprehensive baseline for search interest, data was also cataloged four months before March 11, 2018. This strategic timeframe selection was crucial for our analysis, as it allowed us to document natural fluctuations and trends in search behavior in the absence of significant global health events. By establishing this pre-pandemic baseline, we were equipped to conduct a more precise and insightful comparison of search behaviors before and after the onset of COVID-19.

When queried, Google Trends supplies the relative interest in a term, which indicates the search popularity of that term. The relative interest is a normalized value calculated by dividing the search interest for a term by the total searches made in that timeframe and geographic region and then scaling it from 0 to 100. Google Trends data is normalized for each term separately, so comparing the actual mean difference (∆x̅) among terms is not necessarily informative. Nonetheless, the sign of the mean difference is highly pertinent, as it reports the direction of change. Positive mean differences indicated that search interest increased, and negative values suggest a decrease. The effect size calculated using Cohen’s d reflects the size 

of an increase or decrease in search interest (34). No data points were missing for any terms. Analysis was conducted to understand how search interest changed over time. The data was divided into the baseline, pre-, and post-pandemic periods. The baseline dataset included search interest between January 1, 2018, and March 10, 2018; the pre-pandemic timeframe was set from the weeks of March 11, 2018, to March 8, 2020, and the post-pandemic data was constrained by the weeks of March 15, 2020, to March 13, 2022. To determine whether a significant difference existed in the mean between the two periods, a two-tailed Mann-Whitney U test with a continuity correction was used. The mean difference, effect size, and 95% confidence interval values for both measures were reported. Rho values were categorized as small if below 0.3, moderate if between 0.3 and 0.5, and large if exceeding 0.5 (35).

The analysis was conducted in R version 4.2.0 (The R Foundation for Statistical Computing) using the stats and rstatix packages (36,37), with p*<*0.05 indicating significance. Data collection was expedited with the gtrendsR package (38). 

## Results

In total, relative search interest for 14 terms was collected and analyzed (Table 1).

**Table 1:** Mean (x̅) and standard deviation (SD) of searches for each term in the era before the pandemic, immediately prior to the pandemic start, and following the pandemic

**Term**	**Before Pandemic Era (**x̅±SD**)**	**Pre-Pandemic (**x̅±SD**)**	**Post-Pandemic (**x̅±SD**)**
Aural Fullness	53.44±30.75	38.42±25.55	28.84±19.21
Ear Pressure	71.67±3.61	66.01±7.76	64.61±9.92
Headache	67.78±3.49	61.70±4.00	70.61±9.22
Hearing Loss	77.44±4.22	79.81±8.18	74.27±7.36
Migraine Headache	76.22±4.52	69.19±6.47	73.30±5.71
Neck Pain	88.44±3.00	87.70±3.78	87.69±4.92
Otalgia	67.00±7.21	67.65±14.16	62.38±12.98
Phonophobia	30.78±7.74	32.10±11.51	38.73±15.02
Photophobia	58.44±7.40	61.20±12.79	67.42±11.20
Sudden Hearing Loss	59.56±14.99	61.75±12.11	62.64±15.09
Tinnitus	23.78±1.39	22.08±3.02	26.34±8.52
Dizziness	74.67±3.64	72.31±3.83	78.71±6.04
Motion Sickness	55.22±5.74	66.52±12.94	61.07±14.41
Vertigo	79.89±3.48	80.51±4.81	82.21±6.25

A Mann-Whitney U test was used to determine whether a significant difference in mean existed between search interest two years before and after the pandemic. 

Significant changes were found in 11 terms. Increased search interest was observed in six terms (Table 2).

**Table 2:** Two-tailed Mann-Whitney U test between relative search interest two years before and after the pandemic start date, with a mean difference (∆x̅) and the effect size (r) reported

**Symptoms**	**P-Value**	∆x̅** (CI 95%)**	**r (CI 95%)**
Aural Fullness	*p=*0.006	-7.00 (-17.00, -1.00)	0.19 (0.06, 0.31)
Ear Pressure	*p=*0.142	-2.00 (-4.00, 1.00)	0.10 (0.00, 0.24)
Headache	*p<*0.001	7.00 (6.00, 9.00)	0.59 (0.49, 0.67)
Hearing Loss	*p<*0.001	-6.00 (-8.00, -3.00)	0.33 (0.21, 0.45)
Migraine Headache	*p<*0.001	4.00 (3.00, 6.00)	0.35 (0.23, 0.46)
Neck Pain	*p=*0.935	0.00 (-1.00, 1.00)	0.01 (0.00, 0.15)
Otalgia	*p=*0.003	-6.00 (-9.00, -2.00)	0.20 (0.06, 0.34)
Phonophobia	*p=*0.002	6.00 (2.00, 9.00)	0.22 (0.08, 0.35)
Photophobia	*p<*0.001	6.00 (3.00, 10.00)	0.25 (0.12, 0.37)
Sudden Hearing Loss	*p=*0.863	0.00 (-3.00, 4.00)	0.01 (0.00, 0.16)
Tinnitus	*p<*0.001	3.00 (2.00, 5.00)	0.41 (0.29, 0.52)
Dizziness	*p<*0.001	6.00 (5.00, 8.00)	0.55 (0.45, 0.65)
Motion Sickness	*p=*0.021	-4.00 (-8.00, -1.00)	0.16 (0.03, 0.28)
Vertigo	*p<*0.001	2.00 (1.00, 4.00)	0.23 (0.08, 0.36)

Large increases in search interest were observed for headache (r=0.589, p<0.001), dizziness (r=0.554, p<0.001), and tinnitus (r=0.410, p<0.001). Migraine headaches (r=0.35, p<0.001) had a moderate increase. Smaller increases were noted in phonophobia (r=0.22, p=0.002) and vertigo (r=0.23, p<0.001). Decreased search interest was found for five terms. A moderate decrease was recorded for hearing loss (r=0.33, p<0.001). Phonophobia (r=0.22, p=0.002), otalgia (r=0.20, p=0.003), and aural fullness (r=0.19, p=0.006) exhibited small decreases.

Ear pressure (p=0.142), neck pain (p=0.935), and sudden hearing loss (p=0.863) showed no significant changes. When considering the baseline search interest relative to the two years preceding the pandemic, ear pressure demonstrated a small increase in interest (p=0.007, r=0.25), and migraine headache showed a moderate increase (p<0.001, r=0.31) compared to the pre-pandemic timeframe. Motion sickness manifested a small decrease (p=0.003, r=0.27), with all other terms showing no significant change (Table 3).

**Table 3:** Two-tailed Mann-Whitney U test between relative search interest before the pandemic era in the early pandemic era, with mean difference (∆x̅) and effect size (r) reported

**Symptoms**	**P-Value**	∆x̅** (CI 95%)**	**r (CI 95%)**
Aural Fullness	p=0.148	18.00 (-2.00, 36.00)	0.14 (0.01, 0.32)
Ear Pressure	p=0.007	6.00 (2.00, 10.00)	0.25 (0.13, 0.36)
Headache	p<0.001	7.00 (4.00, 9.00)	0.35 (0.20, 0.47)
Hearing Loss	p=0.303	-3.00 (-8.00, 3.00)	-0.10 (-0.22, -0.01)
Migraine Headache	p<0.001	7.00 (3.00, 11.00)	0.31 (0.16, 0.44)
Neck Pain	p=0.414	1.00 (-2.00, 3.00)	0.08 (0.00, 0.23)
Otalgia	p=0.850	-1.00 (-9.00, 7.00)	-0.02 (-0.17, 0.00)
Phonophobia	p=0.842	-1.00 (-8.00, 6.00)	-0.02 (-0.16, 0.00)
Photophobia	p=0.603	-2.00 (-11.00, 5.00)	-0.05 (-0.19, 0.00)
Sudden Hearing Loss	p=0.498	-3.00 (-13.00, 7.00)	-0.06 (-0.25, 0.00)
Tinnitus	p=0.082	2.00 (0.00, 4.00)	0.16 (0.05, 0.27)
Dizziness	p=0.092	2.00 (0.00, 5.00)	0.16 (0.01, 0.33)
Motion Sickness	p=0.003	-9.00 (-16.00, -3.00)	-0.27 (-0.41, -0.10)
Vertigo	p=0.724	0.00 (-3.00, 2.00)	-0.03 (-0.20, 0.00)

## Discussion

**Fig 1:**
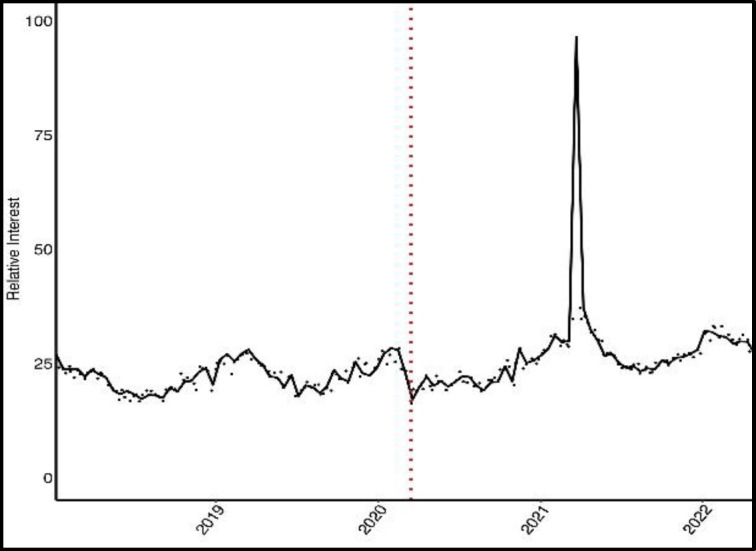
Scatter plot for relative search interest in tinnitus. A spline function illustrates general trends, with the dotted red line indicating the pandemic start date

The search interest for most migraine and migraine-related symptoms increased, including dizziness, headache, aural fullness, and tinnitus (Figure 1).

Some possible explanations for this observed increase might be an increase in people experiencing these symptoms, efforts to be informed about the pandemic (39), and media coverage (25). Many of the symptoms that showed an increase in search interest were strongly associated with COVID-19 infection (40-42). For instance, search interest for headaches and migraine headaches rose, as both were prevalent and widely reported symptoms of COVID-19 infection (40). It is believed that these headaches were caused directly by the virus by raising temperatures, promoting the activity of proinflammatory cytokines, sensitizing the meningeal periphery, or activating the trigeminovascular system (43,44). Headache was also linked to anosmia or ageusia, two highly publicized symptoms (45,46). Therefore, the increased reports of headache in conjunction with media coverage could have led to headache having the most significant increase in search interest.

Dizziness often accompanies viral infection (41), a phenomenon consistent with the observed large increase in search interest. COVID-19 may impair the brainstem and vestibulocollic arc, which impairs communication on the arc to provoke dizziness (47), although respiratory distress, hypoglycemia, or other physiologic statuses may indirectly contribute (48). Therefore, the precise mechanism of action, which suggests cases of dizziness increased in response to the pandemic, may underlie this increase in search interest. Tinnitus also exhibited a large increase in search interest and was widely associated with COVID-19 infection or vaccination (49,50). Proposed mechanisms include damage to cochlea outer hair cells or inflammation of cochleae, which is mediated by viral entry via ACE2, which is abundant in neuronal cells (51). Should the virus be transmitted nasally, its spike protein may facilitate passage through the blood-brain barrier, where it may upregulate proinflammatory mediators and enter brain parenchyma, causing damage that may manifest as hearing complications (51). COVID-19 infection does not necessarily cause new presentation of tinnitus and may instead exacerbate existing tinnitus (52). The increase in search interest for tinnitus may be attributed to the reported cases associated with vaccination or infection (42). Searches for vertigo showed a modest increase in search interest, and while the association between viral infection and vertigo is in contention, associations have been demonstrated in vaccination (53,54). Viral spike proteins may access and disable cochlear hair cells, vestibular hair cells, or afferent auditory or vestibular nerve cells (55), which may cause vertigo. Vertigo was associated with COVID-19, but a mild increase in search interest may be a testament to the uncertainties that cloud the precise mechanism; this same observation can be extended to searches for dizziness.

Search interest also decreased for a select number of symptoms. Cases of hearing loss were published, with authors suggesting associations with COVID-19 infection (51,56) and presenting reasonable mechanisms (56). Yet hearing loss maintains a contentious relationship with viral infection, with studies positing that the increase in hearing loss was not statistically significant (57,58), not adequately supported with audiological testing (59), or exhibited a decreased incidence (60). Sudden hearing loss showed no significant fluctuation in search interest, which is consistent with these studies.

Aural fullness was another symptom that demonstrated a mild decrease in search interest. While this symptom was frequently reported in infected children (50), it generally had consistently lower incidence rates and was not widely investigated in the literature (6,18). This limited coverage in literature and media may have contributed to shifting attention towards more popular symptoms, such as fatigue, difficulty thinking, or loss of smell and taste (61). Notably, less frequently used terms, such as anosmia and ageusia, were found to have search patterns that mirrored media coverage (62). Aural fullness overlaps with other symptoms, such as otalgia, leading to depression in search interest for this specific term.

These results show that Google Trends is a powerful tool and has supported multiple studies by offering data with high validity in salient topics (63). Investigating search trends may yield clinically useful information and may contribute towards the predictive modeling of symptoms, aid in clinical decision-making and management, and help in clinical diagnoses. Google Trends is subject to several limitations. Terms that the public is less familiar with are less likely to be searched, such as in the case of headache, when compared to phonophobia. The data is also highly sensitive to the precise wording used, as demonstrated by the significant decrease observed for hearing loss, which was not mirrored in searches for sudden hearing loss. The former term is ubiquitous, which may have contributed to the considerable increase in searches. Google Trends does not necessarily indicate actual reported or diagnosed cases of a symptom. It is also subject to public perception, media coverage, and user understanding or knowledge. Finally, increases were observed when comparing the pre-COVID era to the two years preceding the pandemic. This demonstrates the inherently dynamic nature of public engagement, information, and interest in medical information. Variables in search behavior not only represent genuine interest in symptoms, but also a complex interplay of factors, including seasonal health concerns, health news, and changes in societal awareness.

## Conclusion

Otologic symptoms were critical in the detection and diagnosis of COVID-19. As infections increased during the pandemic, searches for common symptoms also increased significantly. This increase may be due to more reports of otologic symptoms, people’s desire to stay informed, or media trends. Google Trends effectively captured rises and declines in search interest, making it a valuable epidemiological tool that provides insight into the public perception and understanding of the pandemic.
